# Novel Antiviral and Antibacterial Activities of *Hibiscus schizopetalus*

**DOI:** 10.3390/antibiotics9110756

**Published:** 2020-10-30

**Authors:** Riham A. El-Shiekh, Usama Ramadan Abdelmohsen, Hossam M. Ashour, Rehab M. Ashour

**Affiliations:** 1 Department of Pharmacognosy, Faculty of Pharmacy, Cairo University, Cairo 11562, Egypt; Riham.adel@pharma.cu.edu.eg; 2 Department of Pharmacognosy, Faculty of Pharmacy, Deraya University, New Minia City 61111, Egypt; Usama.ramadan@mu.edu.eg; 3 Department of Pharmacognosy, Faculty of Pharmacy, Minia University, Minia 61519, Egypt; 4 Department of Integrative Biology, College of Arts and Sciences, University of South Florida, St. Petersburg, FL 33701, USA; 5 Department of Microbiology and Immunology, Faculty of Pharmacy, Cairo University, Cairo 11562, Egypt

**Keywords:** *Hibiscus schizopetalus*, antioxidant, antiviral, antimicrobial, anthocyanins, LC-MS

## Abstract

*Hibiscus schizopetalus* (Dyer) Hook.f. (Malvaceae) is an ornamental plant. The aim was to investigate its antimicrobial and antioxidant activities. In vitro antiviral, antibacterial, and antioxidant activities of the 70% ethanolic extract (Et-E) of the aerial parts of the plant were determined. The Dichloromethane Fraction (DCM-F) and the *n*-Butanol Fraction (Bu-F) were assessed using Liquid chromatography–mass spectrometry (LC-MS). The DCM-F showed higher antiviral activities against Coxsackie B4 (CoxB4) viruses (IC_50_ = 64.13 µg/mL) and adenoviruses (IC_50_ = 54.88 µg/mL) than acyclovir (IC_50_ = 72.79 µg/mL for CoxB4 viruses; IC_50_ = 91.92 µg/mL for adenoviruses). The DCM-F showed higher anti-*helicobacter pylori* activity (MIC = 3.9 µg/mL) than clarithromycin (MIC = 1.95 µg/mL). The DCM-F inhibited Herpes Simplex Virus (HSV) Type I (IC_50_ = 29.85 µg/mL) and HSV Type II (IC_50_ = 74.17 µg/mL). The Bu-F showed higher anti-mycobacterial activity (MIC = 7.81 µg/mL) than isoniazid (MIC = 0.24 µg/mL) and higher antibacterial activity against *methicillin-resistant Staphylococcus aureus* (MRSA)(MIC = 7.81 µg/mL) than vancomycin (MIC = 3.9 µg/mL). Antioxidant assays included total antioxidant capacity (TAC), 2,2′-azino-bis-3-ethylbenzthiazoline-6-sulphonic acid (ABTS), 2,2-diphenyl-1-picryl-hydrazyl (DPPH), and iron reducing power. The Bu-F showed the highest antioxidant activity. Chemical profiles were analyzed using HPLC-HR–ESI–MS to identify the metabolites responsible for these biological activities. We identified more than 60 metabolites that belong to anthocyanins, flavonoids, phenolics, terpenes, sterols, and fatty acids. In conclusion, *Hibiscus* *schizopetalus* is endowed with metabolites that could be used against viruses and antibiotic-resistant bacteria. They can also be potent antioxidants.

## 1. Introduction

Antimicrobial resistance to classical antibiotics is a major problem that negatively impacts treatments of common infectious diseases caused by fungi, bacteria, viruses, and parasites. In order to overcome this major problem, natural products offer a viable alternative [1].

*Hibiscus* is a member of Malvaceae family and is cultivated globally as an ornamental and medicinal plant [2]. It is typically distributed in tropical and subtropical regions. *Hibiscus* species have long been known for their economic and therapeutic importance [3]. *Hibiscus schizopetalus* (Dyer) Hook.f.; commonly known as coral Hibiscus; is a shrub that has highly attractive solitary, hanging, and bright red flowers [4,5]. It is indigenous to tropical East Africa [4] and is commonly known as coral *Hibiscus* or fringed *Hibiscus*. *H. schizopetalus* leaves have had a traditional use in treating spermatorrhea. Its flowers have been applied to the scalp as hair tonics. Its fruits have been used in the management of urinary tract problems caused by endocrinological ailments. Different parts of the plant have been used in the treatment of cold, cough, and fever [2,6]. Previous pharmacological studies suggested hypolipidemic, analgesic, hypoglycemic, antipyretic, and anti-inflammatory actions for *H. schizopetalus* but there are no studies that established its activity against the list of bacteria and viruses included in this study [7]. The major constituents of *H. schizopetalus* are anthocyanins, sterols, triterpenes, and phenolics [3,7,8].

Previous studies on other *Hibiscus* species such as *Hibiscus sabdariffa* and *Hibiscus rosa sinensis* flowers reported activities against *methicillin-resistant Staphylococcus aureus* (MRSA) [9,10]. This has not been established for *H. schizopetalus*. Finding new antibacterial and antiviral medications has become very important nowadays in order to be able to face the escalating problem of bacterial resistance. Natural agents, known for lower toxicity and higher effectiveness, are potential alternatives to more toxic and less effective treatments. Antiviral drugs act through inhibiting viral fusion, integration, protease, uncoating, nucleic acid synthesis, and viral release [11].

Newly discovered natural medications that can be used for the treatment and prophylactic of bacterial and viral infections can be viable and safer alternatives to traditional medications. These biologically active metabolites identified from natural products may offer several advantages over synthetic ones including lower toxicity, more biodegradability, and lower costs. Plants are a rich source of bioactive compounds that can lead to the development of new, natural drugs that can have novel mechanisms. This is especially important given the spread of antimicrobial resistance.

The aim of the study was to assess the antiviral, antibacterial, and antioxidant activities of *H. schizopetalus*. In order to accomplish these aims, we tested the 70% ethanolic extract, the methylene chloride fraction, and the *n*-butanol fraction. In addition, their chemical profiles were analyzed by HPLC–HR–ESI–MS. These studies are important in the development of new promising anti-infective agents.

## 2. Results

### 2.1. Cytotoxicity Assay

The 50% cytotoxic concentration (CC_50_) of the 70% ethanolic extract (Et-E), the Dichloromethane Fraction (DCM-F), and the *n*-Butanol Fraction (Bu-F) were 230.80, 325.92, and 288.45 μg/mL, respectively, whereas the maximum non-toxic concentration (MNTC) on Vero cells was estimated to be 78.12 ug/mL. This was used for testing the antiviral activities of *H. schizopetalus*.

### 2.2. Antiviral Activity

The antiviral activity of Et-E was tested using three different protocols. Results are reported using the half maximal inhibitory concentration (IC_50_) (Table 1). At a concentration of 78.12 ug/mL, the Et-E significantly inhibited adenoviruses and Coxsackie B4 (CoxB4) viruses in protocol C by 72.94% and 54.24%, respectively compared to acyclovir inhibitions of 61.35% and 53.9%, respectively. In contrast, mild to weak antiviral activity was observed against HSVI, HSVII, and HAV viruses. The Et-E showed weak activity with protocols A and B. Thus, protocol C was selected for further investigations.

Using protocol C, Et-E, DCM-F, and Bu-F (Table 1 and Table 2) were further examined. DCM-F showed the highest antiviral activity against adenoviruses and CoxB4 viruses, which was higher than Et-E and acyclovir. The DCM-F inhibited adenovirus by an IC_50_ of 54.88 µg/mL, compared to acyclovir’s IC_50_ of 91.92 µg/mL. The DCM-F inhibited CoxB4 virus by an IC_50_ of 64.13 µg/mL compared to acyclovir’s IC_50_ of 72.79 µg/mL. Additionally, the DCM-F inhibited HSVI virus (IC_50_ = 29.85 µg/mL), compared to acyclovir’s IC_50_ of 26.99 µg/mL. DCM-F inhibited HSVII (IC_50_ of 74.17 µg/mL), compared to acyclovir’s IC_50_ of 68.60 µg/mL. DCM-F showed no activity against hepatitis A virus.

### 2.3. Activity against Helicobacter Pylori

The DCM-F showed the highest antibacterial activity against *Helicobacter pylori* with an MIC90 of 2.9 µg/mL and MIC of 3.9 µg/mL, compared to clarithromycin’s MIC90 of 0.7 µg/mL and MIC of 1.95 µg/mL. The anti-*H. pylori* activity of the DCM-F (MIC 3.9 µg/mL) was higher than Bu-F (MIC 15.63 µg/mL), and Et-E (MIC 62.5 µg/mL). Full results are presented in Table 3.

### 2.4. Anti-Mycobacterial Activity

The Bu-F showed the highest antibacterial activity against *Mycobacterium tuberculosis* (*M. tuberculosis)* with MIC90 of 5.6 µg/mL and MIC of 7.81 µg/mL, compared to isoniazid’s MIC90 of 0.04 µg/mL and MIC of 0.24 µg/mL. The Et-E and DCM-F showed much weaker activities. The results are presented in Table 3.

### 2.5. Activity against *Methicillin-Resistant Staphylococcus aureus* *(MRSA*)

The Bu-F showed the highest anti-MRSA activity than other tested samples with MIC90 of 3.7 µg/mL and MIC of 7.81 µg/mL, compared to vancomycin’s MIC90 of 2.23 µg/mL and MIC of 3.9 µg/mL. MRSA was resistant to Et-E and its DCM-F. The results are presented in Table 3.

### 2.6. Antioxidant Activity

The total antioxidant activities of Et-E, DCM-F, and Bu-F were assessed. The Bu-F showed the highest activity followed by the Et-E, and then DCM-F. The Bu-F showed the highest activity percent 2050.5 expressed as a ascorbic acid equivalent in TAC. Bu-F also showed potent scavenging activity by ABTS assay (151.48 µg/mL) and DPPH assay (271.68 µg/mL) as compared to ascorbic acid’s 57.76 µg/mL for ABTS and 19.5 µg/mL for DPPH. Using the iron reducing power assay, Bu-F showed a good reduction power (114.47 µg/mL) compared to ascorbic acid (22 µg/mL). Results are shown in Table 4.

### 2.7. HPLC–HR–ESI–MS

**Figure 1:**
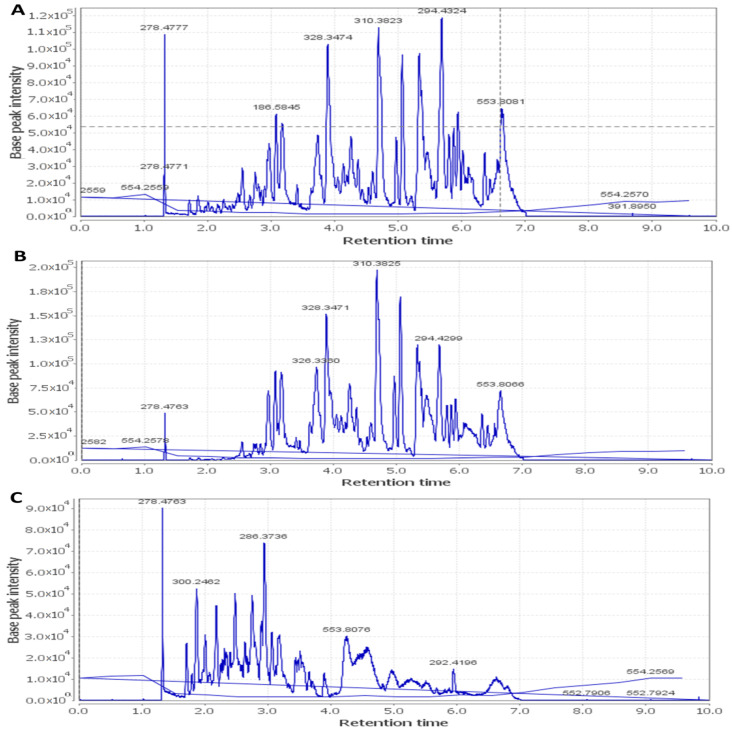
Total ion chromatogram of *Hibiscus schizopetalus* recorded in negative ionization mode. (**A**) Ethanolic extract (Et-E); (**B**) Dichloromethane fraction (DCM-F); (**C**) n-Butanol fraction (Bu-F).

**Figure 2:**
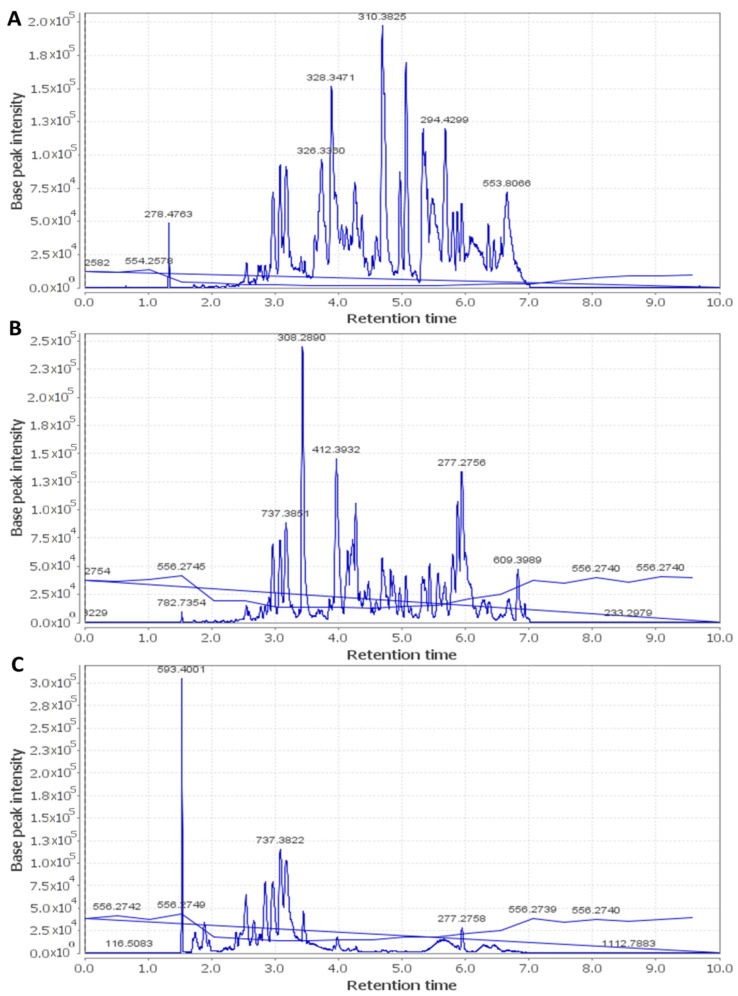
Total ion chromatogram of *Hibiscus schizopetalus* recorded in positive ionization mode. (**A**) Ethanolic extract (Et-E); (**B**) Dichloromethane faction (DCM-F); (**C**) n-Butanol fraction (Bu-F). 2.0 × 10^5^

**Figure 3:**
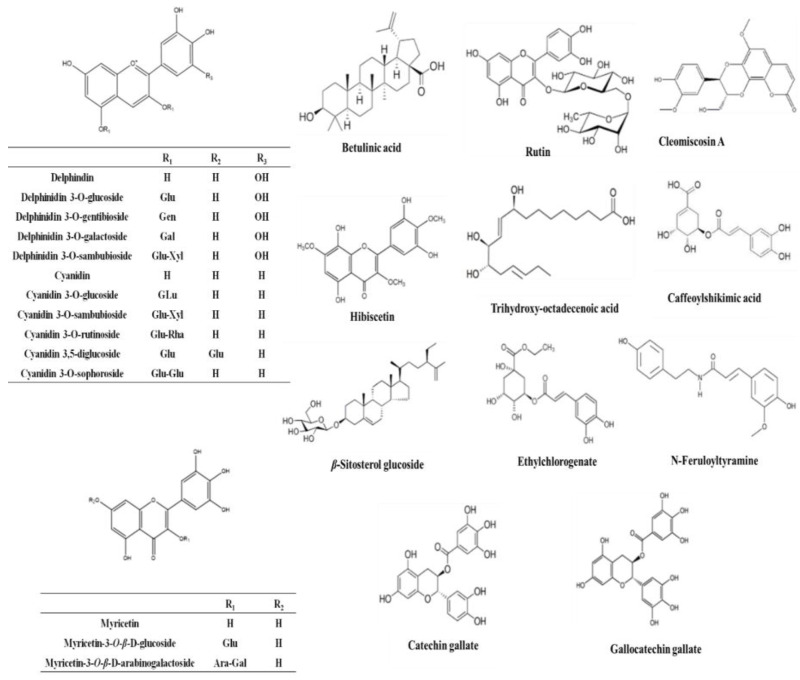
Structures of representative compounds detected in the *Hibiscus schizopetalus* LC-MS profile.

Metabolic profiling using LC–HR–ESI–MS for dereplication purposes [12] led to the identification of more than 60 metabolites and bioactive compounds belonging to different classes such as anthocyanins, flavonoids, phenolic acids, terpenes, sterols, and fatty acids. The detected phytochemicals were tentatively identified by searching DNP and METLIN (Table 5 and Figure 1, Figure 2 and Figure 3).

Regarding anthocyanins, a mass peak at *m/z* 286.17791 (suggested formula C_15_H_10_O_6_) was identified as cyanidin. The mass ion peaks at *m/z* 232.49362 and 264.26644 for the corresponding molecular formulas C_13_H_12_O_4_ and C_10_H_16_O_8_, were dereplicated as hibicuslide C and hibiscus acid hydroxyethyldimethylether, respectively. These were previously reported in *H. sabdariffa* [13]. Furthermore, the suggested molecular formulas C_26_H_28_O_16_ and C_26_H_28_O_15_ with mass ion peaks at 596.71462 and 580.76147 were characterized as delphinidin 3-sambubioside and cyanidin 3-sambubioside, respectively. Both were previously detected in *H. sabdariffa* [14]. In addition to the aforementioned anthocyanins, several others were tentatively identified by comparing their masses with the reported masses [13,15].

Phenolic and flavonoid classes of compounds such as kaempferol-3-O-glucuronic acid methyl ether and gallocatechin gallate were identified from the mass ion peaks at *m/z* 476.01280 and 458.43484, and corresponded to the molecular formulas C_18_H_18_O_14_ and C_22_H_18_O_11_, respectively. This is consistent with previous observations in the genus *Hibiscus* [16]. Another mass ion peak at *m/z* 302.28999 (for the predicted formula C_15_H_10_O_7_) was dereplicated as quercetin. This was previously reported in other *Hibiscus* species [17,18].

A coumarinolignoid, namely cleomiscosin A, with the molecular formula C_20_H_18_O_8_, was characterized from the mass ion peak at *m/z* 386.26719. This was previously reported from the root bark of *H. syriacus* [19]. Similarly, a triterpenoid, namely hydroxy taraxeryl acetate, was detected with the mass ion peak at *m/z* 484.95016, and corresponded to the molecular formula C_32_H_52_O_3_, which was earlier reported in *Hibiscus* species [20]. Moreover, sterols and fatty acids were also characterized in the extract as *β*-sitosterol glucoside and trihydroxy-octadecenoic acid dereplicated from ion peaks at *m/z* 576.74035 and 330.354081, respectively with the proposed molecular formula of C_35_H_60_O_6_ and C_18_H_34_O_5_, respectively.

## 3. Discussion

Medicinal plants and their phytoconstituents are a well-known source of natural anti-infective agents. *H. sabdarrifa* extracts were reported to exhibit potent activity against HSVI [21]. To the best of our knowledge, there are no studies about the antiviral activity of *H. schizopetalus.* In this study, the Et-E of *H. schizopetalus* showed high activity against adenovirus and CoxB4 virus (using protocol C) and weak to moderate activity against HSVI, HSVII, and HAV. To further investigate whether certain metabolites can account for this antiviral potential, DCM-F and Bu-F were similarly tested. The DCM-F showed the most potent activity against adenovirus and CoxB4 virus.

The antiviral activity of the Et-E of *H. schizopetalus* may be attributed to synergistic effects of anthocyanins, terpenes, sterols, fatty acids, phenolic acids, and flavonoids as detected by LC-MS-MS. The potent antiviral activity of the nonpolar metabolites such as fatty acids and other lipids in DCM-F was previously reported [22,23,24,25]. Phytosterols inhibit protease, reverse transcriptase, viral replication, and maturation as well as abate cellular oxidative stress, which can contribute to their antiviral activities [24,26]. For example, the in vitro anti-HIV activity of β-sitosterol can be attributed to the inhibition of viral protease, in addition to its immunomodulatory actions [26,27]. Taraxerol acetate, a conjugated triterpene, has also showed anti-HSV type II activity [28]. Other triterpenes such as oleanolic acid and its derivatives have well reported antiviral activities and can be used as vaccine adjuvants [29]. Betulinic acid, a pentacyclic triterpenoid, has also been reported to exhibit antiviral activity through inhibition of protease and inhibition of viral replication [27].

Moreover, fatty acids and their derivatives have been reported to display antiviral activities, such as trihydroxy-octadecenoic acid, which is active against influenza A and B viruses [30]. Phenolics such as catechin gallate and gallocatechin gallate are also potent antivirals and can inhibit nanoparticle-based RNA oligonucleotide [27]. In addition, flavonoids such as quercetin, myricetin, and scutellarein have the ability to inhibit viral fusion, integration, reverse transcription, and replication [27]. In addition to quercetrin and rutin, coumarinolignoids, such as cleomiscosin, are antivirals that can inhibit reverse transcriptase, viral entry, viral replication, and can potentially regulate the immune response [31,32]. Finally, anthocyanins such as cyanidin, delphinidin, and pelargonidin are antivirals that can disrupt the viral activity and interfere with the viral fusion and attachment [11].

The antibacterial activity of Et-E and its fractions were examined against *H. pylori*, *M. tuberculosis,* and *MRSA*. The DCM-F showed the most potent anti-*H. pylori* activity followed by Bu-F, and Et-E, as compared to clarithromycin (MIC 1.95 µg/mL). *H. sabdariffa* aqueous extracts were also shown to possess anti-*H. pylori* activity with MIC values ranging from 9.18 to 16.68 µg/mL [33]. In this study, the Bu-F showed the highest anti-mycobacterial and anti-MRSA activity. The anti-MRSA effect has been previously reported for the flowers of other *Hibiscus* species, such as *H. sabdariffa* and *H. rosa sinensis* [9,10]. The antibacterial activity of the Bu-F could be attributed to the flavonoid component, which could inhibit DNA gyrase and impact the bacterial cell wall [34]. Anthocyanins were also shown to have bacteriostatic activity [11].

In antioxidant studies with an ascorbic acid control, methanolic extracts of flowers and leaves of *H. schizopetalus* showed significantly lower values than ascorbic acid when assessed by DPPH radical scavenging and nitric oxide antioxidant assay [35]. In this study, the Bu-F showed the highest antioxidant activity followed by the Et-E and the DCM-F; all three had significantly higher antioxidant activities compared with the ascorbic acid control. The work of Wong et al. on the leaves and flowers of *H. schizopetalus* showed mixed results, with unimpressive antioxidant values when using total phenolic content (TPC) and free radical scavenging (FRS), but better values when using ferrous ion chelating ability and lipid peroxidation inhibition [7]. This ambiguity might be due to the lack of proper controls (no ascorbic acid control or any other controls) and/or the difference in anthocyanin content. The antioxidant activity in the Bu-F could be attributed to gossypitrin [36]. Moreover, anthocyanins such as delphinidin can enhance antioxidant activity by increasing the levels of glutathione, free-radical scavenging, decreasing ROS and lipid peroxidation [11,37].

It is noteworthy that this is the first detailed metabolic profiling of *H. schizopetalus.* Among various metabolites tentatively identified herein, only 22-Hydroxy taraxeryl acetate was isolated before from the leaves in addition to cyanidin 3-sambubioside, quercetin, and rutin, which were previously detected in leaves and stems of *H. schizopetalus* [3,8].

## 4. Materials and Methods

### 4.1. Plant Material and Chemicals

The aerial parts (leaves, stems, and flowers) of *H. shizopetalus* were collected during the flowering stage in May 2019, from Orman Botanic Garden, Giza, Egypt and authenticated by Mrs. Therese Labib, Botanical specialist and consultant at Orman and Qubba Botanic Gardens. A voucher specimen (26 November 2019) was deposited in the Museum of the Department of Pharmacognosy, College of Pharmacy, Cairo University, Egypt.

We used analytical grade solvents (ethanol, dichloromethane, and *n*-butanol) for plant extraction and fractionation (El-Gomhouria Co., Cairo, Egypt).

The chemicals 2,2′-azino-*bis*-3-ethylbenzthiazoline-6-sulphonic acid (ABTS), 2,2-diphenyl-1-picryl-hydrazyl (DPPH), ascorbic acid, and trichloro acetic acid were obtained from Aldrich Chemicals, U.S.A. Sodium phosphate, ammonium molybdate, potassium persulfate, potassium ferricyanide, and ferric chloride were purchased from El-Nasr Company for Pharmaceutical Chemicals, Egypt. Phosphate buffer was purchased from Bio diagnostic, Egypt. HPLC-grade Acetonitrile and methanol were purchased from SDFCL Fine-Chem Limited, Mumbai, India.

### 4.2. Extraction and Fractionation

The powdered plant material (1 kg) was extracted in 70% ethanol (5 × 2.5 L) till exhaustion using an Ultra-Turrax^®^ T25 homogenizer (Janke & Kunkel IKA-Lab., Staufen, Germany). The ethanolic extract (Et-E) was evaporated to dryness under reduced pressure (90 g). An aliquot of the dry residue (45 g) was suspended in water (300 mL) and partitioned, successively, with dichloromethane (3 × 500 mL) and *n*-Butanol saturated with water (3 × 500 mL). The solvents were evaporated under reduced pressure to give the DCM-F (29 g), and the Bu-F (11 g). The extract and fractions were kept in the desiccator over anhydrous CaCl_2_.

### 4.3. Cytotoxicity Assay

Vero cells were isolated from kidney epithelial cells extracted from African green monkey (*Cercopithecus aethiops*). The colorimetric assay of 3-[4-dimethylthiazol-2-yl]-2,5-diphenyl tetrazolium bromide (MTT) was performed spectrophotometrically using a Perkin-Elmer ELISA reader (HTS 7000 plus) at 540 nm [38,39]. The minimum concentration producing alterations in cell morphology is the MNTC. The percentage of cytotoxicity was calculated as [(A − B)/A] × 100, where A and B were the optical densities (OD) of untreated and treated cells, respectively [40]. The CC_50_ is the concentration that reduced the cell viability by 50% compared to untreated controls. The CC_50_ was determined by plotting different concentrations of extracts on the *X* axis and cell viability on *Y* axis, where cell viability (%) = Mean OD/Control OD × 100%.

### 4.4. Cell Culture and Virus

The Vero cell line (Cercopithecus aethiops normal adherent kidney epithelial cells, CCL-81) was cultured in RPMI 1640 medium (Gibco, Tunisia) complemented with fetal bovine serum (10% *v*/*v*), L-glutamine (2 mM), penicillin (100 U/mL), and streptomycin (100 μg/mL) and incubated at 37 °C in a humidified atmosphere with 5% CO_2_. Adenovirus, CoxB4, herpes simplex virus type 1 (HSV-I), herpes simplex virus type 2 (HSV-II), and hepatitis A virus (HAV) were provided by the Laboratory of Virology, Science Way for Scientific Research and Consultations, Faculty of Medicine, Al-Azhar University, Egypt.

### 4.5. Antiviral Activity

Viruses and the Vero cell cultures were treated with the minimum non-toxic concentration of the Et-E using protocols A, B, and C as indicated below [41,42,43,44].

Protocol A (virus pretreatment): testing the extract’s virucidal activity by exposing the viruses to the extract for one hour at 37 °C, followed by addition of the mixture (100 μL) to the cells cultured in a microtiter plate.

Protocol B (cell pretreatment): testing the viral entry into the host cells via blocking the attachment to the cell surface by incubating the extract on Vero cells for one hour followed by the addition of the virus.

Protocol C (post-infection treatment): testing the effect of the extract on viral replication through virus incubation for one hour on Vero cells followed by the addition of the extract.

The viability of infected and non-infected cells was determined using the absorbance values of formazan in an MTT assay. The IC_50_ is the concentration required to inhibit 50% of the viral growth. The selectivity index (SI) equals the ratio of the CC_50_ to the 50% antiviral concentration (IC_50_); SI = CC_50_/IC_50_. Means of six determinations were calculated.

### 4.6. Activity against Helicobacter pylori

Activity against *Helicobacter pylori* was assessed using a micro-well dilution method [45]. *H. pylori* inocula (ATCC 43504) were prepared (10^6^ CFU/mL). The subsequent twofold dilutions (0.24–125 μg/mL) of the tested samples were prepared in DMSO. Clarithromycin and DMSO were used as positive and negative controls, respectively. In a 96-well plate, 40 μL of the growth medium (brain heart infusion with 10% fetal bovine serum) was added then 10 μL of inoculum and finally 50 μL of the tested samples. The plate was incubated for 3 days at 37 °C in 5% O_2_, 10% CO_2_, and 85% N_2_ atmosphere. Subsequently, 40 μL of MTT (0.5 mg/mL) was added and incubated for 30 min. The percentage inhibition of bacterial growth by different concentrations of each sample was calculated: % inhibition = [(Abs_control_−Abs_sample_) × 100/Abs_control_]. The minimum concentration required for the inhibition of bacterial growth (MIC) and the concentration required for inhibition of 90% of the bacterial growth (MIC90) were determined from the corresponding dose-response curve. The MTT assay was done using an automatic BioTek plate reader at 620 nm. 

### 4.7. Anti-Mycobacterial Activity

The anti-mycobacterial activity against *M. tuberculosis (M. Tub ATCC 27294)* was detected using the Microplate Alamar Blue Assay (MABA) [46]. Isoniazid was used as a reference drug. Serial dilutions (0.24–125 μg/mL) of the tested samples and isoniazid, dissolved in DMSO, were prepared in the microplate then 0.1 mL of a *M. tuberculosis* inoculum (10^5^ CFU/mL) was added. Plates were incubated at 37 °C for 4 days, then 20 μL of Alamar Blue solution and 12.5 μL of 20% Tween 80 were added. The plates were re-incubated at 37 °C for 24 h. The color intensity was measured using an ELISA microplate reader at 590 nm. The experiment was done in triplicates.

### 4.8. Activity against *Methicillin-Resistant Staphylococcus aureus* *(MRSA)*

The anti-MRSA activity *(MRSA ATCC 27294)* was assessed using the microdilution method in the presence of tetrazolium salts [47]. Vancomycin was used as a reference drug. In a 96-well microplate, 40 μL of the growth medium (brain heart infusion; BHI), plus 10% Fetal Bovine Serum (FBS), 10 μL of the inoculum (10^6^ CFU/mL), and 50 μL of the sample dilutions (0.24–125 μg/mL) were added. The plates were incubated at 37 °C for 24 h. To each well, 40 μL of XTT were added and incubated for 1 h in the dark at 37 °C. For each well, the color intensity was measured at 492 nm.

### 4.9. Antioxidant Activity

*A*. *Total antioxidant capacity assay (TAC).*

The antioxidant activity of the samples was assessed using the phosphomolybdenum method [48,49]. Absorbance was measured at 695 nm after incubation at 95 °C for 150 min. The TAC was expressed in ascorbic acid equivalents (AAE) (μmol/g of extract).

*B*. *2,2′-azino-bis-3-ethylbenzthiazoline-6-sulphonic acid (ABTS) radical scavenging assay.*

The ABTS radical scavenging assay was used to assess the free radical scavenging activity of the *Hibiscus* extract and its fractions [48,49]. Measurements were carried out at 734 nm. The ABTS radical scavenging ability was expressed as IC50 (mg/mL). Ascorbic acid was used as a standard.

*C*. *2,2-diphenyl-1-picryl-hydrazyl (DPPH) radical scavenging assay.*

The anti-oxidant activity was measured as previously described [49] Absorbance was measured at 517 nm. Ascorbic acid was used as standard. IC50 was determined using the equation [(Ac – As)/Ac] × 100. Ac is the control absorbance (DPPH solution without test sample) while As is the sample absorbance (DPPH solution in addition to the extract or the standard). The IC50 is the concentration that produces 50% inhibition of absorbance at the indicated wavelength.

*D*. *Reducing power assay.*

The reducing power was assessed by the evaluating the ability of the samples to change potassium ferricyanide to potassium ferrocyanide. Absorbance was measured at 700 nm [48,49]. The EC50 is the effective concentration (mg/mL) acquired from linear regression analysis with an absorbance of 0.5. The effective concentration providing an absorbance of 0.5 was calculated. Ascorbic acid was used as a standard. 

Experiments for antioxidants were done in triplicates and means were calculated.

### 4.10. HPLC–HR–ESI–MS

The crude methanolic extract and fractions for mass spectrometry were prepared at 1 mg/mL. Metabolomic analysis of the samples was done using LC-HR-ESI-MS [50,51]. We used an Acquity Ultra Performance Liquid Chromatography system that was connected to a Synapt G2 HDMS quadrupole time-of-flight hybrid mass spectrometer (Waters, Milford, CT, USA). Positive and negative ESI ionization modes were used to perform the high-resolution mass spectrometry coupled with a spray voltage of 4.5 kV, capillary temperature of 320 °C, and a mass range from *m/z* 150–1500. 

The data were imported and analyzed using MZmine 2.20 [52]. The mass ion peaks in positive and negative ionization mode data sets were dereplicated against the databases of the Dictionary of Natural Products.

### 4.11. Statistical Analysis

Data analysis was done using analysis of variance (ANOVA). The significance of differences between means was done using Duncan’s multiple range tests at *p* < 0.05.

## 5. Conclusions

*H. schizopetalus* is endowed with a lot of chemical compounds (more than 60 metabolites) that could be clinically used in fighting viruses, such as adenoviruses, HSVI, and CoxB4. They can also be used as effective therapies against antibiotic resistant pathogens such as *M. tuberculosis*, MRSA, and *H. pylori*. Finally, they can be potent antioxidants that can protect cells from free radical damage in cancer, heart diseases, and other serious health conditions.

## Figures and Tables

**Table 1:** Antiviral activity of *Hibiscus schizopetalus.*

Name of Virus	Protocol A		Protocol B		Protocol C	
Adenovirus	Et-E	39.66	Et-E	58.40	Et-E	72.94 ^c^
					DCM-F	78.78 ^d^
					Bu-F	33.41 ^a^
	Acyclovir	41.95	Acyclovir	59.90	Acyclovir	61.35 ^b^
CoxB4	Et-E	33.25	Et-E	22.50	Et-E	54.24 ^b^
					DCM-F	60.90 ^c^
					Bu-F	33.96 ^a^
	Acyclovir	41.50	Acyclovir	36.32	Acyclovir	53.90 ^b^
HSV I	Et-E	47.57	Et-E	25.90	Et-E	62.67 ^a^
					DCM-F	91.80 ^c^
					Bu-F	80.79 ^b^
	Acyclovir	75.20	Acyclovir	42.33	Acyclovir	95.48 ^c^
HSV II	Et-E	9.49	Et-E	27.79	Et-E	30.84 ^b^
					DCM-F	51.75 ^c^
					Bu-F	1.78 ^a^
	Acyclovir	23.05	Acyclovir	46.44	Acyclovir	55.69 ^d^
HAV	Et-E	0.55	Et-E	0	Et-E	12.10 ^b^
					DCM-F	18.28 ^c^
					Bu-F	5.60 ^a^
	Acyclovir	21.60	Acyclovir	0	Acyclovir	30.47 ^d^

The percentage of inhibition was determined at a concentration of 78.12 µg/mL. Different superscripts (a, b, c & d) for a given value within a column indicate significant difference at *p* < 0.05 using Duncan’s Multiple Range test (DMRT). Ethanolic Extract (Et-E); Dichloromethane Fraction (DCM-F); *n*-Butanol Fraction (Bu-F); Coxsackie (CoxB4); Herpes Simplex Virus Type I (HSV-I); Herpes Simplex Virus Type II (HSV-II); Hepatitis A Virus (HAV). Protocol A: Virus pretreatment; Protocol B: Cell pretreatment; Protocol C: Post-infection treatment.

**Table 2:** IC_50_ (µg/mL) and selectivity index (SI) of *Hibiscus schizopetalus* by protocol C.

Virus	Parameter	Sample			Standard
		Et-E	DCM-F	Bu-F	Acyclovir
Adenovirus	IC_50_	58.89 ^b^	54.88 ^a^	108.31 ^d^	91.92 ^c^
	SI	3.8	5.93	2.66	8.34
CoxB4	IC_50_	72.0 ^b^	64.13 ^a^	107.30 ^c^	72.79 ^b^
	SI	3.20	5.08	2.68	10.53
HSV I	IC_50_	66.86 ^d^	29.85 ^b^	50.94 ^c^	26.99 ^a^
	SI	3.45	10.91	5.66	28.42
HSVII	IC_50_	126.65 ^c^	74.17 ^b^	-	68.60 ^a^
	SI	1.82	4.39		11.18
HAV	IC_50_	-	168.67 ^b^	-	95.40 ^a^
	SI		1.93		8.04

The half maximal inhibitory concentration (IC_50_) was calculated in µg/mL; selectivity index (SI). Different superscripts (a, b, c & d) for a given value within a column indicate significant difference at *p* < 0.05 using Duncan’s Multiple Range test (DMRT). Ethanolic Extract (Et-E); Dichloromethane Fraction (DCM-F); *n*-Butanol Fraction (Bu-F). Coxsackie Virus (CoxB4); Herpes Simplex Virus Type I (HSV-I); Herpes Simplex Virus Type II (HSV-II); Hepatitis A Virus (HAV).

**Table 3:** Antibacterial activity of *Hibiscus schizopetalus.*

Sample/Standard	*Helicobacter pylori*		*Mycobacterium tuberculosis*		*MRSA*	
	MIC90	MIC	MIC90	MIC	MIC90	MIC
Et-E	22.7 ^c^	62.5 ^c^	98.6 ^d^	125 ^d^	17.56 ^b^	31.25 ^c^
DCM-F	2.9 ^a^	3.9 ^a^	12.3 ^c^	15.63 ^c^	44.4 ^c^	62.5 ^d^
Bu-F	13.7 ^b^	15.63 ^b^	5.6 ^b^	7.81 ^b^	3.7 ^a^	7.81 ^b^
Clarithromycin	0.7 ^a^	1.95 ^a^	-	-	-	-
Isoniazid	-	-	0.04 ^a^	0.24 ^a^	-	-
Vancomycin	-	-	-	-	2.23 ^a^	3.9 ^a^

MIC and MIC90 values were calculated in μg/mL Ethanolic Extract (Et-E); Dichloromethane Fraction (DCM-F); *n*-Butanol Fraction. (Bu-F); Methicillin-resistant *Staphylococcus aureus* (MRSA). Different superscripts (a, b, c & d) for a given value within a column indicate significant difference at *p* < 0.05 using Duncan’s Multiple Range test (DMRT).

**Table 4:** Antioxidant activity of *Hibiscus schizopetalus.*

Sample/Standard	TAC%	ABTS	DPPH	Iron Reducing Power
	(AAE)	IC_50_ (µg/mL)		EC_50_ (µg/mL)
Et-E	1850.83 ^b^	710.51 ^c^	473.29 ^c^	391.45 ^c^
DCM-F	800.16 ^c^	819.51 ^d^	1118.92 ^d^	528.0 ^d^
Bu-F	2050.50 ^a^	151.48 ^b^	271.68 ^b^	114.47 ^b^
Ascorbic acid	-	57.76 ^a^	19.50 ^a^	22 ^a^

Different superscripts (a, b, c & d) for a given value within a column indicate significant difference at *p* < 0.05 using Duncan’s Multiple Range test (DMRT). Ascorbic Acid Equivalent (AAE); Ethanolic Extract (Et-E); Dichloromethane Fraction (DCM-F); *n*-Butanol Fraction (Bu-F).

**Table 5:** Metabolites of *Hibiscus schizopetalus* (identified by HPLC–HR–ESI–MS).

Metabolite	Molecular Formula	Exact Mass	Et-E	DCM-F	Bu-F
**Anthocyanins**					
**Cyanidin**	C_15_H_10_O_6_	286.177914	+	++	++
**Cyanidin *O*-rhamnoside**	C_21_H_20_O_10_	432.385735	+	+++	++
**Cyanidin *O*-galactoside/Cyanidin *O*-glucoside**	C_21_H_20_O_11_	448.956000	+++	++	++
**Cyanidin 3-sambubioside**	C_26_H_28_O_15_	580.761473	+	+++	+
**Cyanidin *O*-rutinoside**	C_27_H_30_O_15_	594.399238	+	+	+++
**Cyanidin di-*O*-glucoside**	C_27_H_30_O_16_	610.308112	+	++	+
**Cyanidin 3-(digalloylglucoside)**	C_35_H_28_O_19_	752.646798	+	++	+
**Cyanidin 3-(acetylgalloylgalactoside)**	C_30_H_26_O_16_	642.403723	+	+++	+
**Gossypetin**	C_15_H_10_O_8_	318.287501	+	+++	+
**Cyanidin 3-(*O*-succinoyl-glucopyranoside)**	C_25_H_24_O_14_	548.577150	+	++	+
**Cyanidin 3-(oxalylglucoside)**	C_23_H_20_O_14_	520.445023	+	+++	+
**Cyanidin 3-(cinnamoylglucoside)**	C_30_H_26_O_12_	578.566536	+	+++	+
**Cyanidin 3-(malonylxyloside)**	C_23_H_20_O_13_	504.916039	+	+++	+
**Cyanidin 3-(xylosylarabinoside)**	C_25_H_26_O_14_	550.464496	+	+++	+
**Gossypetin glucoside (Gossypitrin)/Myricetin-*O*-glucoside**	C_21_H_20_O_13_	480.861466	+	+	+++
**Gossypetin glucuronide**	C_21_H_18_O_14_	494.876248	+	+	+++
**Delphinidin**	C_15_H_10_O_7_	302.192080	+	+	+++
**Delphinidin galactoside/Delphinidin glucoside**	C_21_H_20_O_12_	464.370561	+	+	+++
**Delphinidin 3-sambubioside (Hibiscin)**	C_26_H_28_O_16_	596.714621	++	++	+++
**Delphinidin neohesperidoside/Delphinidin rutinoside**	C_27_H_30_O_16_	610.371790	+	+	+++
**Dimethyl-delphinidin-glucosyl acetate**	C_25_H_26_O_13_	534.494206	+	+++	+
**Delphinidin 3-gentiobioside**	C_27_H_29_O_17_	626.404530	+	+++	+
**Hibicuslide C**	C_13_H_12_O_4_	232.493625	+	+	+
**Hibiscetin**	C_15_H_10_O_9_	334.283647	+	++	+
**Hibiscetin glucoside**	C_21_H_20_O_14_	496.438349	+	+++	+
**Hibiscus lactone (Hydroxycitric acid lactone)**	C_6_H_6_O_7_	190.177052	+	+++	+
**Hibiscus acid hydroxyethyldimethylether**	C_10_H_16_O_8_	264.266440	*+*	*+*	*+++*
**Hibiscus acid hydroxyethylether**	C_8_H_12_O_8_	236.156126	+	+	+++
**Malvidin**	C_17_H_14_O_7_	330.249624	*+*	*+++*	*+*
**Malvidin *O*-(coumaroylglucoside)**	C_32_H_30_O_14_	638.416871	*+*	*+*	*+++*
**Petunidin**	C_16_H_12_O_7_	316.269633	*+*	*+++*	*+*
**Pelargonidin**	C_15_H_10_O_5_	270.221390	*+*	*++*	*+*
**Pelargonidin glucoside**	C_21_H_20_O_10_	432.385735	*+*	*+++*	*+*
**Flavonoids/Phenolic acids**					
**Kaempferol**	C_15_H_10_O_6_	286.194597	+	+	+
**Kaempferol rhamnoside**	C_22_H_22_O_10_	446.215495	+	-	+++
**Kaempferol *O*-glucuronide**	C_21_H_18_O_12_	462.214878	+	-	+++
**Kaempferol-3-*O*-glucuronic acid methyl ether**	C_18_H_18_O_14_	476.012800	+	+	+
**Kaempferol *O*-rutinoside**	C_27_H_30_O_15_	594.284269	+	-	+++
**Kaempferitrin**	C_27_H_30_O_14_	578.749072	+	-	+++
**Scutellarein rhamnopyranoside glucopyranoside**	C_27_H_30_O_15_	594.284027	+	-	+++
**Myricetin**	C_15_H_10_O_8_	318.287992	+	+++	+
**Myricetin *O*-arabinogalactoside**	C_26_H_28_O_17_	612.594615	+	+	+++
**Quercetin**	C_15_H_10_O_7_	302.289996	+	+++	+
**Quercitrin/Kaempferol glucoside**	C_21_H_20_O_11_	448.956137	+	++	+++
**Rutin**	C_27_H_30_O_16_	610.658348	+	-	+++
**Apigenin**	C_15_H_10_O_5_	270.221390	+	+++	+
**Apigenin-*O*-acetylxyloside**	C_23_H_22_O_11_	474.215366	+	-	+++
***O*-Caffeoyl-hydroxycitric acid**	C_15_H_14_O_11_	370.137822	+	-	+++
***O*-Caffeoylshikimic acid**	C_16_H_16_O_8_	336.317691	+	+++	+
**Catechin gallate**	C_22_H_18_O_10_	442.440382	+	+++	+
**Gallocatechin gallate**	C_22_H_18_O_11_	458.434848	+	+++	+
**Ethylchlorogenate**	C_18_H_22_O_9_	382.143465	+	+++	+
**Trimethylhydroxycitric acid**	C_9_H_14_O_8_	250.149487	+	-	+++
**Coumaroylquinic acid**	C_16_H_18_O_8_	338.281091	+	-	+++
**N-Feruloyltyramine**	C_18_H_19_NO_4_	312.252784	*+*	*+*	*+++*
**Cleomiscosin A**	C_20_H_18_O_8_	386.267194	+	+++	-
**Cleomiscosin C/D**	C_21_H_20_O_9_	416.424465	+	+++	-
**Sterols/Terpenes/Fatty acids**					
***β*-Sitosterol**	C_29_H_50_O	414.405049	+	+++	-
***β*-Sitosterol glucoside**	C_35_H_60_O_6_	576.740355	+	+++	-
**Hydroxy taraxeryl acetate**	C_32_H_52_O_3_	484.950167	+	+++	-
**Betulinic acid/Oleanolic acid**	C_30_H_48_O_3_	456.920845	+	+++	-
**Mansonone H**	C_15_H_14_O_4_	258.253920	+	+++	-
**Palmitic acid**	C_16_H_32_O_2_	256.1772030	+	+++	-
**Linolenic acid**	C_18_H_30_O_2_	278.474709	+	+++	-
**Linoleic acid**	C_18_H_32_O_2_	280.3010786	+	+++	-
**Trihydroxy-octadecenoic acid**	C_18_H_34_O_5_	330.3540819	+	+++	-
**Stearic acid**	C_18_H_36_O_2_	284.3896759	+	+++	-
**Docosanoic acid**	C_22_H_44_O_2_	340.35180228	+	+++	-
**Docosanedione**	C_22_H_42_O_2_	338.31881164	+	+++	-
**Methyldocosadienoic acid**	C_23_H_42_O_2_	350.31891388	+	+++	-

(-) absent, (+) low, (++) moderate, (+++) high.

## References

[1] Khameneh B., Iranshahy M., Soheili V., Bazzaz B.S.F. (2019). Review on plant antimicrobials: A mechanistic viewpoint. Antimicrob. Resist. Infect. Control.

[2] Chan E.W., Wong S., Chan H. (2016). A review on the phytochemistry and pharmacology of two *Hibiscus* species with spectacular flower colour change: *H. tiliaceus* and *H. mutabilis*. Int. J. Pharmacogn. Phytochem. Res..

[3] Abdelhafez O.H., Othman E.M., Fahim J.R., Desoukey S.Y., Pimentel-Elardo S.M., Nodwell J.R., Schirmeister T., Tawfike A., Abdelmohsen U.R. (2020). Metabolomics analysis and biological investigation of three Malvaceae plants. Phytochem. Anal..

[4] Pal S., Sarkar J., Bhattacharya S., Biswas M. (2011). Thi layer chromatographic studies ad assessme t of a ti-i flammatory effect of *Hibiscus schizopetalus* leaf extracts in rats. Pharmacologyonline.

[5] Wong S.K., Lim Y.Y., Chan E.W.C. (2009). Antioxidant properties of *Hibiscus*: Species variation, altitudinal change, coastal influence and floral colour change. J. Trop. For. Sci..

[6] Zahid H., Rizwani G.H. (2016). Antimicribial efficacy of *Hibiscus schizopetalus* (Mast) Hook. Hamdard Med..

[7] Wong S., Chan E.W., Chan H. (2016). A review on the phytochemistry and pharmacology of two lesser-known *Hibiscus* species: *H. taiwanensis* and *H. schizopetalus*. Int. J. Pharmacogn. Phytochem. Res..

[8] Jose E., Vijayan K. (2006). New taraxerane esters from *Hibiscus schizopetalus* leaves. Indian J. Chem..

[9] Hayati Z., Yulia W., Karmil T.F., Azmy A. Anti-bacterial activity of rosella flowers extract (Hibiscus sabdariffa linn) in inhibiting bacterial growth methicillin-resistant Staphylococcus aureus. Proceedings of the Annual International Conference, Syiah Kuala University-Life Sciences & Engineering Chapter.

[10] Arullappan S., Zakaria Z., Basri D.F. (2009). Preliminary screening of antibacterial activity using crude extracts of *Hibiscus rosa sinensis*. Trop. Life Sci. Res..

[11] Pour P.M., Fakhri S., Asgary S., Farzaei M.H., Echeverria J. (2019). The signaling pathways, and therapeutic targets of antiviral agents: Focusing on the antiviral approaches and clinical perspectives of anthocyanins in the management of viral diseases. Front. Pharmacol..

[12] Ito T., Masubuchi M. (2014). Dereplication of microbial extracts and related analytical technologies. J. Antibiot..

[13] Salem M.A., Michel H.E., Ezzat M.I., Okba M.M., L-Desoky A.M.E., Mohamed S.O., Ezzat S.M. (2020). Optimization of an Extraction solvent for angiotensin-converting enzyme inhibitors from hibiscus sabdariffa l. based on its UPLC-MS/MS metabolic profiling. Molecules.

[14] Obouayeba A., Djyh N., Diabate S., Djaman A., N’guessan J., Kone M., Kouakou T. (2014). Phytochemical and antioxidant activity of Roselle (Hibiscus sabdariffa L.) petal extracts. Res. J. Pharm. Biol. Chem. Sci..

[15] Rasheed D.M., Porzel A., Frolov A., el Seedi H.R., Wessjohann L.A., Farag M.A. (2018). Comparative analysis of Hibiscus sabdariffa (roselle) hot and cold extracts in respect to their potential for α-glucosidase inhibition. Food Chem..

[16] Da-Costa-Rocha I., Bonnlaender B., Sievers H., Pischel I., Heinrich M. (2014). Hibiscus sabdariffa L.-A phytochemical and pharmacological review. Food Chem..

[17] Sharma M.C., Smita S., Kohli D.V. (2010). Phytochemical and anti-ulcer investigations of 95% ethanolic-benzene-chloroform leaf extract of Hibiscus tiliaceus Linn. in Albino rat model. Ann. Biol. Res..

[18] Barve V.H., Hiremath S., Pattan S., Pal S. (2010). Phytochemical and pharmacological evaluation of *Hibiscus mutabilis* leaves. J. Chem. Pharm. Res..

[19] Yun B.-S., Lee I.-K., Ryoo I.-J., Yoo I.-D. (2001). Coumarins with monoamine oxidase inhibitory activity and antioxidative coumarino-lignans from hibiscus s yriacus. J. Nat. Prod..

[20] Salem M.Z., Olivares-Pérez J., Salem A. (2014). Studies on biological activities and phytochemicals composition of Hibiscus species—A review. Life Sci. J..

[21] Torky Z.A., Hossain M.M. (2017). Pharmacological evaluation of the hibiscus herbal extract against herpes simplex virus-type 1 as an antiviral drug in vitro. Int. J. Virol..

[22] Thormar H., Isaacs C.E., Brown H.R., Barshatzky M.R., Pessolano T. (1987). Inactivation of enveloped viruses and killing of cells by fatty acids and monoglycerides. Antimicrob. Agents Chemother..

[23] Rezanka T., Siristova L., Sigler K. (2009). Sterols and triterpenoids with antiviral activity. Anti-Infect. Agents Med. Chem. (Former. Curr. Med. Chem.—Anti-Infect. Agents).

[24] Khwaza V., Oyedeji O.O., Aderibigbe B.A. (2018). Antiviral activities of oleanolic acid and its analogues. Molecules.

[25] Librán-Pérez M., Pereiro P., Figueras A., Novoa B. (2019). Antiviral activity of palmitic acid via autophagic flux inhibition in zebrafish (Danio rerio). Fish Shellfish Immunol..

[26] Parvez M.K., Alam P., Arbab A.H., Al-Dosari M.S., Alhowiriny T.A., Alqasoumi S.I. (2018). Analysis of antioxidative and antiviral biomarkers β-amyrin, β-sitosterol, lupeol, ursolic acid in Guiera senegalensis leaves extract by validated HPTLC methods. Saudi Pharm. J..

[27] Islam M.T., Sarkar C., El-Kersh D.M., Jamaddar S., Uddin S.J., Shilpi J.A., Mubarak M.S. (2020). Natural products and their derivatives against coronavirus: A review of the non-clinical and pre-clinical data. Phytother. Res..

[28] Kuljanabhagavad T., Suttisri R., Pengsuparp T., Ruangrungsi N. (2009). Chemical structure and antiviral activity of aerial part from Laggera pterodonta. J. Health Res..

[29] Masullo M., Pizza C., Piacente S. (2017). Oleanane derivatives for pharmaceutical use: A patent review (2000–2016). Expert Opin. Ther. Pat..

[30] Nagai T., Kiyohara H., Munakata K., Shirahata T., Sunazuka T., Harigaya Y., Yamada H. (2002). Pinellic acid from the tuber of Pinellia ternata Breitenbach as an effective oral adjuvant for nasal influenza vaccine. Int. Immunopharmacol..

[31] Yadav D.K., Meena A., Srivastava A., Chanda D., Khan F., Chattopadhyay S. (2010). Development of QSAR model for immunomodulatory activity of natural coumarinolignoids. Drug Des. Dev. Ther..

[32] Teponno R.B., Kusari S., Spiteller M. (2016). Recent advances in research on lignans and neolignans. Nat. Prod. Rep..

[33] Hassan S.T., Berchová K., Majerová M., Pokorná M., Švajdlenka E. (2016). In vitro synergistic effect of *Hibiscus sabdariffa* aqueous extract in combination with standard antibiotics against Helicobacter pylori clinical isolates. Pharm. Biol..

[34] Donkor S., Larbie C., Komlaga G., Emikpe B.O. (2019). Phytochemical, antimicrobial, and antioxidant profiles of *Duranta erecta* L. parts. Biochem. Res. Int..

[35] Zahid H., Rizwani G.H., Shareef H., Ali S.T. (2014). Antioxidant and urease inhibition activity of methanol extract of Hibiscus schizopetalus (Mast) Hook. J. Pharmacogn. Phytochem..

[36] François-Haugrin F., Monan M., Nossin E., Smith-Ravin J., Marcelin O. (2016). Antioxidant activity of an isomer of gossypitrin (gossypetin-3’-O-glucoside) isolated in the petals of *Talipariti elatum* Sw., and determination of total phenolic content of the total flower. J. Pharmacogn. Phytochem..

[37] Masheta D.Q., Al-Azzawi S.K. (2018). Antioxidant and Anti-Inflammatory Effects of Delphinidin on Glial Cells and Lack of Effect on Secretase Enzyme. IOP Conference Series: Materials Science and Engineering.

[38] Elaissi A., Rouis Z., Salem N.A.B., Mabrouk S., ben Salem Y., Salah K.B.H., Aouni M., Farhat F., Chemli R., Harzallah-Skhiri F. (2012). Chemical composition of 8 *Eucalyptus* species’ essential oils and the evaluation of their antibacterial, antifungal and antiviral activities. BMC Complement. Altern. Med..

[39] Takeuchi H., Baba M., Shigeta S. (1991). An application of tetrazolium (MTT) colorimetric assay for the screening of anti-herpes simplex virus compounds. J. Virol. Methods.

[40] Goodger J.Q., Woodrow I.E. (2011). α, β-Unsaturated monoterpene acid glucose esters: Structural diversity, bioactivities and functional roles. Phytochemistry.

[41] Gong E.Y. (2013). Antiviral Methods and Protocols.

[42] Ocazionez R.E., Meneses R., Torres F.Á., Stashenko E. (2010). Virucidal activity of Colombian Lippia essential oils on dengue virus replication in vitro. Memórias Inst. Oswaldo Cruz.

[43] Gescher K., Kühn J., Hafezi W., Louis A., Derksen A., Deters A., Lorentzen E., Hensel A. (2011). Inhibition of viral adsorption and penetration by an aqueous extract from *Rhododendron ferrugineum* L. as antiviral principle against herpes simplex virus type-1. Fitoterapia.

[44] Okba M.M., el Gedaily R.A., Ashour R.M. (2017). UPLC–PDA–ESI–qTOF-MS profiling and potent anti-HSV-II activity of *Eucalyptus sideroxylon* leaves. J. Chromatogr. B.

[45] Bonacorsi C., Raddi M.S.G., Carlos I.Z., Sannomiya M., Vilegas W. (2009). Anti-Helicobacter pylori activity and immunostimulatory effect of extracts from Byrsonima crassa Nied (Malpighiaceae). BMC Complement. Altern. Med..

[46] Lu Y., Zheng M., Wang B., Fu L., Zhao W., Li P., Xu J., Zhu H., Jin H., Yin D. (2011). Clofazimine analogs with efficacy against experimental tuberculosis and reduced potential for accumulation. Antimicrob. Agents Chemother..

[47] Tunney M.M., Ramage G., Field T.R., Moriarty T.F., Storey D.G. (2004). Rapid colorimetric assay for antimicrobial susceptibility testing of Pseudomonas aeruginosa. Antimicrob. Agents Chemother..

[48] El-Shiekh R.A., Ashour R.M., Abd El-Haleim E.A., Ahmed K.A., Abdel-Sattar E. (2020). *Hibiscus sabdariffa* L.: A potent natural neuroprotective agent for the prevention of streptozotocin-induced Alzheimer’s disease in mice. Biomed. Pharmacother..

[49] Chaouche T.M., Haddouchi F., Ksouri R., Atik-Bekkara F. (2014). Evaluation of antioxidant activity of hydromethanolic extracts of some medicinal species from South Algeria. J. Chin. Med. Assoc..

[50] Abdelmohsen U.R., Cheng C., Viegelmann C., Zhang T., Grkovic T., Ahmed S., Quinn R.J., Hentschel U., Edrada-Ebel R. (2014). Dereplication strategies for targeted isolation of new antitrypanosomal actinosporins A and B from a marine sponge associated-Actinokineospora sp. EG49. Mar. Drugs.

[51] Gamaleldin N.M., Bakeer W., Sayed A.M., Shamikh Y.I., El-Gendy A.O., Hassan H.M., Horn H., Abdelmohsen U.R., Hozzein W.N. (2020). Exploration of chemical diversity and antitrypanosomal activity of some red sea-derived actinomycetes using the OSMAC approach supported by LC-MS-based metabolomics and molecular modelling. Antibiotics.

[52] Tawfike A., Attia E.Z., Desoukey S.Y., Hajjar D., Makki A.A., Schupp P.J., Edrada-Ebel R., Abdelmohsen U.R. (2019). New bioactive metabolites from the elicited marine sponge-derived bacterium Actinokineospora spheciospongiae sp. nov. AMB Express.

